# Potential of focused ultrasound in epilepsy surgery

**DOI:** 10.1186/2050-5736-3-S1-O29

**Published:** 2015-06-30

**Authors:** Nathan Fountain, Phil Tseng, Mark Quigg, Robert Dallapiazza, Jeff Elias

**Affiliations:** 1 University of Virginia, Charlottesville, VA, United States

## Background/introduction

The minimally invasive nature of FUS makes it an excellent candidate technology for epilepsy surgery. The limitation of currently available FUS systems is that the lesion must be deep in the brain to avoid bone heating. Thus, it is best suited for “subcortical” epilepsies, i.e. epilepsy resulting from lesions below the cortex. It is also desirable that the lesion be relatively small volume to avoid long treatment times and not be in eloquent cortex. Previous reports of seizure freedom after laser ablation of hypothalamic hamartomas and radiofrequency ablation of single subcortical nodules have been reported.

Thus, we designed the Focused Ultrasound Subcortical Epilepsy (FUSE) study with these principles in mind.

## Methods

The FUSE study is an open label safety and feasibility pilot study. It will enroll 15 subjects with subcortical lesions as the cause of medically refractory epilepsy, including hypothalamic hamartomas, periventricular nodular heterotopia, focal “cortical” dysplasia that is sufficiently far from the skull, and hamartomas of tuberous sclerosis. The main outcome is safety and feasibility of ablating the lesion but secondary outcome includes seizure reduction. Subjects will undergo a single MR guided FUS treatment session. Subjects may be sedated since they are likely to be in the MRI for 2-4 hours. A targeting model will be created before the procedure. Low temperature heating will first insure proper localization of energy at the lesion, and then a high temperature ablation will be performed. If this study is successful, then it could change the standard treatment of hypothalamic hamartoma which now currently is primarily treated by laser ablation or open surgery. If the study is successful in treating periventricular nodules then it could be transformative for these patients because there is currently no common method of surgical treatment.

FUS has a high potential to provide effective ablation of subcortical lesions causing epilepsy. Future developments will likely allow a larger treatment envelope and expand its use to other epilepsies. If it is a low risk procedure then it could shift the risk-benefit ratio towards empiric treatment of lesions and help avoid intracranial monitoring and its attendant risks.

